# INTERVENTIONS TARGETING LONELINESS AND ISOLATION AMONG LONG-TERM CARE RESIDENTS: A SYSTEMATIC REVIEW

**DOI:** 10.1093/geroni/igac059.2587

**Published:** 2022-12-20

**Authors:** Audrieanna Raciti, Amy Lyons, Yu-Ping Chang

**Affiliations:** Buffalo, New York Mills, New York, United States; University at Buffalo, Buffalo, New York, United States; University at Buffalo, Buffalo, New York, United States

## Abstract

The impact of loneliness and social isolation on health is a significant concern among people residing in long term care. Researchers have begun to design and test interventions to lessen the burden of these impacts on the individual as well as our already strained healthcare system. The purpose of this review is to synthesize types and outcomes of interventions designed to reduce loneliness and isolation among long term care residents. Six databases: CINAHL, PUBMED, Cochrane, Web of Science, PsycInfo, and Embase were searched to identify studies leveraging the use of various interventions for alleviating feelings of loneliness and isolation within long term care residents. Multiple keywords were used including loneliness, social isolation, long-term care, nursing home, treatment, and strategies. Articles were screened if they were published between 2012 and present. Twenty studies were included in this systematic review. All studies have a small sample size. Only four studies were randomized controlled trials. Interventions included laughter therapy, adaptive sports, social robotics, horticultural therapy, spirituality, electronic cognitive behavioral therapy, music therapy, virtual reality, expressive arts, telephone discussions, video conferencing, peer mentoring, and animal assisted therapy. The majority of the interventions demonstrate a significant improvement in alleviating feelings of loneliness and social isolation, while some interventions also show improvement in quality of life and physical ability. However, some studies have a high attrition rate. Future research is needed to utilize a more rigorous design with a large sample size. Participant engagement should be considered as part of the intervention activities.

